# A self-guided Internet-delivered intervention for adults with ADHD: a protocol for a randomized controlled trial

**DOI:** 10.1016/j.invent.2021.100485

**Published:** 2021-11-20

**Authors:** Robin Maria Francisca Kenter, Astri J. Lundervold, Tine Nordgreen

**Affiliations:** a Department of Clinical Psychology, Faculty of Psychology, University of Bergen, Christies gate 12, 5015 Bergen, Norway; b Department of Biological and Medical Psychology, Faculty of Psychology, University of Bergen, Jonas Lies vei 91, 5009 Bergen, Norway; c Division of Psychiatry, Haukeland University Hospital, Haukelandsbakken 15, 5009 Bergen, Norway; d Department of Global Public Health and Primary Care, Faculty of Medicine, University of Bergen, Årstadveien 17, Block D, 5009 Bergen, Norway

**Keywords:** Internet-delivered self-help, Attention deficit hyperactivity disorder, Randomized controlled trial, Inattention, Hyperactivity, Quality of life

## Abstract

**Background:**

Attention-deficit/hyperactivity disorder (ADHD) in adulthood, with an estimated prevalence of 2–3%, is associated with several challenges in daily life functioning. The availability of evidence-based psychological interventions for adults with ADHD is still poor. Interventions delivered over the Internet on smartphones or personal computers may help to increase the availability of effective psychological interventions. The primary aim of this randomized controlled trial is to examine the efficacy of a self-guided Internet-delivered intervention on severity levels of ADHD symptomatology and quality of life.

**Methods:**

We aim to include 118 participants with a self-reported ADHD diagnosis in a randomized controlled trial with two arms: 1) self-guided Internet-delivered intervention for coping with ADHD (*N* = 59); 2) self-guided online psychoeducation (control group, N = 59). After 3 months, the control group will be given access to the intervention. The primary clinical outcomes are inattention and quality of life. Secondary clinical outcomes are hyperactivity, stress and depression. Measures will be obtained at three time points: before (baseline), immediately after (8 weeks) and 3 months after the intervention. Uptake, usage, adherence and satisfaction will be explored.

**Discussion:**

This RCT will provide valuable information on the clinical effectiveness of an Internet-delivered intervention for adults with ADHD. This study is, to our knowledge, one of the first randomized control trials that investigates the effects of a self-guided Internet-delivered psychological intervention in a fairly large group of adults with ADHD.

**Trial registration:**

ClinicalTrials.gov, Identifier NCT04726813, January 27, 2021.

## Highlights

• The availability of evidence-based psychological interventions for adults with ADHD is poor.
• Interventions-delivered interventions may help to increase the availability of effective interventions for this group.
• This paper describes a study protocol for a randomized controlled trial that examines the efficacy of MyADHD.
• MyADHD was developed in cooperation with adults with an ADHD diagnosis.
• The intervention is expected to improve symptoms of inattention and quality of life.

## 1 Introduction

Attention Deficit Hyperactivity Disorder (ADHD) is a neurodevelopmental disorder characterized by pervasive symptoms of inattention and/or hyperactivity/impulsiveness that persist across different situations (Edition, 2013). Longitudinal studies have shown that at least 60% of children diagnosed with ADHD will continue to show symptoms into adulthood with a severity level that requires treatment (Faraone et al., 2006; Franke et al., 2018; Kessler et al., 2005a). With an estimated prevalence as high as 2–3%, ADHD is a common disorder in adulthood.

It is important to provide effective treatment to adults with ADHD, as the disorder is associated with problems related to the regulation of behaviour, cognition and emotions and often causes a wide range of challenges in daily life functioning (Franke et al., 2018). Availability of appropriate psychological interventions for adults with ADHD should thus have a great impact on the daily life functioning at an individual and societal level.

Pharmacological treatment is commonly a first choice of treatment for adults with ADHD. Although effective on a group basis, it is acknowledged that many show intolerance to medication, lack of response or residual impairments and/or side effects such as reduced sleep and appetite (Spencer et al., 2000). Due to this, non-pharmacological treatment options are increasingly demanded by adults with ADHD (Solberg et al., 2019; Vidal et al., 2013).

Increased availability and use of non-pharmacological treatment options are part of official policy (Nutt et al., 2007; Gibbins and Weiss, 2007), but so far, psychological treatments for ADHD are still not widely available in routine clinical practice. Few adults with ADHD are offered psychological treatment following completion of neuropsychiatric assessment and diagnosis, even though long-term studies have underscored its importance (Kooij et al., 2019).

Several psychological interventions are developed for, or adapted to, adults with ADHD in the recent years. Although the evidence base for psychological treatment is still relatively limited, some recent studies based on cognitive behavioral therapy (CBT) (Knouse and Safren, 2010; Anastopoulos et al., 2021; Weiss et al., 2008; Weiss et al., 2012) and dialectical behaviour therapy (DBT) (Hesslinger et al., 2002; Hirvikoski et al., 2011) have shown promising results, both when it comes to reducing ADHD related symptoms and increasing life quality. The focus in these treatments is to enhance executive and organizational skills, improvement of consequence thinking and impulse control as well as emotion regulation skills. Other interventions like goal management training (GMT), have also been investigated as treatment options for adults with ADHD (Dymphie et al., 2017; Braek et al., 2009; Nordby et al., 2021; Jensen et al., 2021). As executive functions, like goal-directed and context appropriate behaviour, are often impaired in adults with ADHD, GMT teaches participants strategies for stopping unwanted behaviours, improving planning of activities and structuring intentions.

In sum, most treatment guidelines to date recommend a combination of pharmacological and non-pharmacological treatment options for adults with ADHD, given that a significant number cannot tolerate, do not respond to, or fail to reach optimal results with medication alone. Psychological interventions like CBT, GMT and DBT for ADHD provide new, compensatory strategies and skills for deficient attention, executive functioning, impulse control and emotion regulation, but they are often not available to adults with ADHD due to barriers such as: limited resources, lack of knowledge and qualified therapists, and other factors such as geographical distance and stigma (Solberg et al., 2019; Weiss et al., 2008).

### 1.1 Internet-delivered interventions for adults with ADHD

Psychological interventions delivered over the Internet may help to overcome these barriers. They have the potential to offer treatment outside of the traditional treatment centres, reach individuals in regions where psychological treatment is not available and offer more flexibility for the patient (Andersson, 2016). Internet-delivered interventions can also provide a cost-effective opportunity to provide 24-h access to information, advice and support. These interventions can be provided with therapist support, so called guided Internet-delivered interventions, or without therapist support, so called self-guided Internet-delivered interventions. Self-guided Internet-delivered interventions often provide daily support in the form of automatic reminders, feedback regarding progress, overcoming obstacles and help with planning daily activities (Titov et al., 2014). These type of interventions can help managing symptoms of ADHD by providing general information, tailored advice, support and skill training via technology (e.g. smartphones, tablets or websites) (Andersson and Titov, 2014). Internet-delivered interventions are expected to be appropriate for adults with ADHD who have insufficient response to medication, who are in need for an add-on treatment option, and/or prefer non-pharmacological treatment options. Some individuals with an ADHD diagnosis already use timers, schedules and reminders, tools that are available on their smartphones (Moëll et al., 2015). However, there is a lack of controlled studies investigating whether self-guided Internet-delivered interventions contribute to improved daily functioning for adults with ADHD.

### 1.2 Objectives

The primary objective of this study is to evaluate the effects of a self-guided Internet-delivered psychological intervention on i) symptoms of inattention and quality of life in adults with ADHD, and ii) symptoms of hyperactivity, stress and depression. These effects will be compared with results obtained in a group of adults with ADHD randomized to a psychoeducation control condition. The secondary objective of the study is to explore user satisfaction with and adherence to the intervention.

### 1.3 Trial design

The planned study will use a randomized controlled trial (RCT) design with two arms, 1) a self-guided Internet delivered psychological intervention, 2) an Internet-delivered psychoeducation control condition. We hypothesize that the psychological intervention is superior to psychoeducation. The study will include two follow-up assessments, at 8 and 12 weeks after baseline measurements to assess longer term effects of the intervention.

## 2 Methods

### 2.1 Study setting and design

The responsible study center is located at the University of Bergen, Norway. All data will be collected using online questionnaires and telephone interviews. Data will be collected from eligible participants in Norway. Participants will be recruited via social media and the Norwegian ADHD organization.

This randomized controlled trial has two arms: arm I is a self-guided Internet-delivered intervention (MyADHD), arm II is a psychoeducation control. Due the nature of the intervention and the comparison, it is impossible to blind people to the condition.

### 2.2 Eligibility criteria

Adults (≥18) with a self-reported ADHD diagnosis are eligible to participate in the study. Participants will be screened for inclusion and exclusion criteria before randomization.

Criteria for inclusion:

a) Age ≥18
b) A self-reported diagnosis of ADHD (date, venue and diagnosing physician)
c) Access to and ability to use a computer, smartphone and the Internet.
d) Current self-reported problems with organizing daily activities
e) Participant is by investigators considered able to follow through the intervention protocol and take part in assessment measures
f) Speaks, writes, and reads Norwegian
g) Participant who is taking prescribed ADHD medication has to be stable on the medication at least four weeks before the study and during the timespan of the study.

Exclusion criteria:

a) Current self-reported diagnosis of severe psychiatric illness such as borderline or antisocial personality disorder, bipolar disorder,
b) Ongoing substance abuse
c) Ongoing suicidal ideation assessed with item 10 on the Montgomery Aasberg Depression Rating Scale (Fantino and Moore, 2009)
d) Ongoing engagement in another psychological treatment

### 2.3 Informed consent and withdrawal from the study

Participants who are eligible to participate in the study, as assessed by a pre-screening questionnaire and a follow-up telephone interview, will have to sign a consent (see questionnaires and participant timeline section). The consent will be signed digitally in a secure portal with BankID (level 4 protection). Participants can end their participation at any time and for any reason. In addition, should the condition of a participant deteriorate, supervising clinicians may choose to end the participation prematurely and direct the participant to official health care services.

### 2.4 Intervention

#### 2.4.1 MyADHD Intervention description and development

The intervention will be delivered via an online secure portal, which is accessible on smartphones, laptops and personal computers. The intervention is a short-term, structured self-guided intervention with modified elements from CBT, DBT and GMT to target specific challenges experienced by adults with ADHD. As ADHD in adulthood affects many psychosocial domains, we expect that a combination of therapeutic techniques from various psychotherapeutic frameworks that already have been examined and have shown promising effects in face-to-face and group therapy, might be more beneficial to adults with ADHD than focusing on one treatment framework alone. CBT for adults with ADHD includes behavioral interventions targeting the practice of compensatory skills and cognitive interventions targeting negative thoughts, avoidance and procrastination. Strategies include prioritization, organization, problem solving and stress management. DBT strategies include emotional regulation, impulse control, self-regulation, self-esteem and self-acceptance (Swales and Heard, 2016), while GMT has a stronger focus on inhibitory control (Stamenova and Levine, 2019). By combining treatment frameworks, it is possible to tailor the intervention to key concern and difficulties reported by adults with ADHD, as shown in a recent publication from our research group (Flobak et al., 2021).

The creation of the content of the modules was done by using the Person-based approach (Yardley et al., 2015) and included: consultation with experts and members of user groups; examination of relevant theory and evidence from previous trials and treatment manuals. The intervention has been iteratively modified for optimization from user perspective. We held several focus group meetings with *N* *=* 12 adult participants with ADHD, and a lived-experience workshop with *N* = 11 adults with ADHD. In the lived experience workshop, we interviewed participants about their experiences with coping with ADHD. This information formed the content of the videos, which are included in the intervention. In the videos, an actor plays out the lived experience in one video (e.g., problems with attention) and in the second video, an actor plays out the coping strategies (Flobak et al., 2021). A pilot study has been performed and shown promising results (Nordby et al., 2021).

The intervention consists of seven training modules that are released weekly for the intervention group participants. The main goals of the intervention are to help participants to obtain improved functioning in daily life activities; offer strategies that will lead to stress reduction, reduce inattention and improve quality of life. The intervention includes: a short introductory chapter (open to everyone), followed by a start module (goal setting) and six different themed optional modules. A brief overview of modules content is shown in Table 1. Each module includes psychoeducation alongside text, audio and video material instructing participants in the use of specific techniques. Participants will be encouraged to complete weekly action plans and symptom questionnaires for each module. Further, modules include case vignettes and lived-experience videos serving to clarify important training principles and help participants make connections between the material and their own experiences. Automatic reminders are sent when a participant does not log on within one day after release of a new module, or not finishes a module within 4 days. Homework is given after modules 2–6, where the participants are asked to continue training in everyday situations on the newly learned skills and to log situations in which the participants succeeded or failed.

**Table 1:** Overview of the intervention in Arm I.

Module^a^	Rationale and content
Obligatory modules	
Information module	Information about the study
1. Start module	Goal setting and practical information about how to use the Internet-delivered intervention.
2. Mindful awareness	Inattention is a core symptom of ADHD. In this module, participants are given information about different aspect of attention and concentration and how to cope with impairment. In this module the participants start training on mindful awareness (“being here and now”) by focusing on their breathing. *Based on DBT*.
3. Inhibition training	Impulsivity and loss of impulse control are common among adults with ADHD. This module consists of exercises focussing on impulse control and goal oriented-directed behaviour (stop, observe, proceed, check). *Based on GMT*
4. Emotional regulation	Emotional instability and brief recurrent states of feelings are common in ADHD. In this module, participants are first informed about modern theories of emotion (primary emotions, relationship cognition-emotion and emotion-behaviour) and then presented exercises to improve emotional analysis (emotions record, emotion diary) and emotional regulation. *Based on DBT*.
5. Planning and organizing daily life	Problems with planning an organizing sequential behaviour often result in situations where adults with ADHD do several different things at the same time, feel pressurized, and end up finishing none of their planned tasks. Adults with ADHD often feel that this behaviour is a result of emotional stress and experience this deficit as stressful in itself. This module presents stress management techniques, how to handle a tendency towards procrastination, breaking down large tasks. *Based on CBT*.
6. Self-acceptance	In this module the focus is on accepting what one cannot change and to be kind to oneself. Psychoeducation about understanding how actions are separate from ones qualities, identifying and acknowledging strengths, practice forgiveness. The goal is to enhance self-compassion. *Based on DBT*.
7. Summary and road ahead	Summary of all the modules and planning for the future

a Features common across all modules: automatic reminders, (homework) exercises, downloadable hand-outs, weekly questionnaires, progress graphs, lived-experience videos.

#### 2.4.2 Psychoeducation control

Participants in the control condition will be assigned to psychoeducation modules (see Table 2) and will receive restricted access to the platform. They can contact or be contacted by the research team if their symptoms levels increase.

**Table 2:** Overview of the psychoeducation control in Arm II.

Module	Rationale and content
Modules	
Information module	Information about the study
Psychoeducation	Prevalence, etiology, symptom description, general advice and strategies for coping with ADHD. Links to patient information websites and apps for mobile phone (calendar, planner etc).

### 2.5 Criteria for discontinuing or modifying allocated interventions

Key stopping rules for patients are a withdrawal of informed consent or unwillingness to further participate in the trial or any factors affecting the patient's well-being. When participants score above 20 on the PHQ-9; express suicidal ideation (a score MADRS-SR ≥ 4); or write concerning messages in the open text fields, they will be contacted by the research team for a screening. Based on clinical evaluation it will be decided whether a participant should continue the intervention.

### 2.6 Outcomes

All outcome questionnaires are self-report scales that participants themselves can fill out via an online secure platform. Assessments are repeated at baseline, 8 weeks, and 12 weeks. An overview of the measurements is giving in Table 3.

**Table 3:** Questionnaires and measures.

	Screening	Baseline	Weekly in intervention a	8 week post-test	3 M follow up
Eligibility criteria					
Diagnosis of ADHD (self-report) - Date - Venue - Diagnosing physician	x				
Access to pc, smartphone or laptop with Internet connection	x				
Understands Norwegian	x				
Current stable use of ADHD medication (self-report) - Yes - No - Type	x				
Ongoing substance abuse (self-report)	x				
Self-reported severe mental illness such as borderline or antisocial personality disorder, bipolar disorder and/or mental retardation	x				
Currently receiving psychological treatment for ADHD	x				
Availability for the next 4 weeks	x				
MADRS-SR (item 10; suicidality)	x	x		x	x
Demographic variables questionnaire - Gender - Age - Educational level - Occupational status - Personal contact information (email, telephone number) - Postal code	x	x			
ASRS		x	x	x	x
AAQol		x		x	x
PHQ-9		x		x	x
GAD-7		x		x	x
PSS		x		x	x
Changes in medication and engagement in other therapeutic interventions questionnaire		x		x	x
Self-report adverse events				x	x

Usability measures					
Use, benefit and understanding of the modules components (questionnaire specifically design, open questions)			x		
CEQ		x		x	x

Participant-generated data					
User login Completed modules Completed questionnaires Completed homework Completed assignments Time spent on reading material Time spent on module/exercise Clicks			x	x	x

Content evaluation					
Participant feedback within the program			x		

a These measures will only be filled out by the intervention group. AAQOL-29: Adult ADHD Quality of Life Scale. ASRS: the adult ADHD self-rating scale. CEQ: credibility and expectancy scale. MADRS: Montgomery And Åsberg Depression Rating Scale. MADRS SR: Montgomery And Åsberg Depression Rating Scale Suicide Rating. PHQ-9: patient health questionnaire 9. PSS: perceived stress scale.

#### 2.6.1 Primary outcome measure

##### 2.6.1.1 The Adult ADHD Self-Rating Scale (ASRS)

The Adult ADHD Self-Rating Scale (ASRS) (Kessler et al., 2005b) includes the 18 symptoms of ADHD described in the diagnostic manual (DSM-5). ASRS is a self-report scale with 18 items, with items included in one of two subscales; one scale assessing symptoms of inattention (9 questions), and one scale assessing symptoms of hyperactivity/impulsivity (9 questions). The response type consists of a 5-point Likert scale with options “Never” (0), “Rarely” (Edition, 2013), “Sometimes” (Faraone et al., 2006), “Often” (Franke et al., 2018) or “Very Often” (Kessler et al., 2005a) giving the scale a total point of 72 for full-scale ASRS and 36 each on the two subscales. Test–retest reliability of the Norwegian translation of ASRS is reported to be 0.88 (Kornør and Hysing, 2011).

#### 2.6.2 Secondary outcome measures

##### 2.6.2.1 Adult ADHD Quality of Life Measure (AAQol)

The AAQoL has 29 items designed to assess health related quality of life during the past two weeks among adults with ADHD (Gjervan and Nordahl, 2010). Each item is rated by participants on a five-point Likert scale. The AAQoL yields a total score (based on all 29 items) and four subscale scores: Life Productivity, Psychological Health, Life Outlook, and Relationships. Internal consistency is acceptable, with Cronbach's alpha >0.70 for all subscales.

##### 2.6.2.2 The perceived stress scale (PSS)

The Perceived Stress Scale (PSS) is a widely used psychological instrument for measuring stress (Cohen et al., 1994). Items were designed to measure stress and how uncontrollable respondents find their lives during the week (Cohen et al., 1983). The PSS version used in this study has 10 items with response alternatives 0 (never) to 4 (very often) and Cronbach's alpha has been reported to be 0.89 (Roberti et al., 2011).

##### 2.6.2.3 The Patient Health Questionnaire-9 (PHQ-9)

The Patient Health Questionnaire-9 (PHQ-9: (Kroenke et al., 2001)) is a self-report tool used to assess the presence and severity of depressive symptoms. Reliability and validity of the tool have indicated that it has sound psychometric properties. Internal consistency of the PHQ-9 has been shown to be high. A study involving two different populations in Norway produced Cronbach alphas of 0.86 and 0.88 (Wisting et al., 2021).

#### 2.6.3 Usage, user satisfaction and engagement

##### 2.6.3.1 Usage

During the intervention, the following measures of activity and compliance will be measured; number of logins; number of finished exercises; total time spent on modules and exercises; and finally, how many pages each participant finished.

User satisfaction is measured by four open-end questions at the end of each of the modules:

1. Were there parts of the module that you experienced as helpful and/or supportive?
2. Were there parts of the module that you experienced as complicated and/or unhelpful?
3. Would you recommend this module to a friend or a family member with similar difficulties as yourself?
4. How useful did you find this module? 1–10

##### 2.6.3.2 The credibility and expectancy scale (CEQ)

CEQ (Devilly and Borkovec, 2000) is a 6-item self-report measure that assesses intervention credibility and expectancy for improvement. The first four items of the scale are rated based on cognitive appraisals about the intervention, while the last two items are rated based on feelings about the intervention. The CEQ has demonstrated good validity and reliability (Devilly and Borkovec, 2000).

##### 2.6.3.3 Engagement

Usage outcomes of interest are attrition, adherence to the intervention and time spent on the intervention.

### 2.7 Participant timeline

#### 2.7.1 Procedure

*Step 1*. *Online anonymous survey*. The survey will take place in an open version of an online platform with no BankID login. The following questions are asked in step 1:

1. Are you 18 years or older?

2. Do you have a diagnosis of ADHD?

3. Do you have time to actively participate in the study over the next 12 weeks?

4. Do you have access to a computer or smartphone with Internet?

5. Are you able to read and write Norwegian?

When not meeting inclusion criteria, participants are rejected access to the intervention. To meet inclusion criteria, the participant will have to answer yes on all the five questions above.

*Step 2*: *Telephone screening*. Eligible participants will be asked to schedule an appointment for screening by telephone with a member of the research team. During the screening they will be asked about the following:

1. Age when diagnosed with ADHD.

2. Venue where the ADHD diagnosis was given.

3. Current symptoms of ADHD.

4. Day-to day functioning (work, school and social).

5. Suicidality (Item 10 MADRS).

6. Symptoms of depression.

7. Symptoms of psychosis.

8. Symptoms of bipolar disorder.

9. Substance use.

10. Current psychological treatment or other psychological treatment needs.

11. Motivation.

12. Name.

13. Email address.

14. National identity number.

When not meeting inclusion criteria, participants are rejected access to the platform. If necessary, they will be referred to other appropriate sources of support. The same procedure will be followed if exclusion from the study is needed.

*Step 3*: Signing consent. Participants who are eligible to participate in the study will have to sign a consent to participate in the study. The consent is digitally signed in a secure online portal with BankID.

*Step 4*: *Randomization*. Participants who are eligible for the study will be randomized with sealed envelopes and assigned to one of two groups: the internet-delivered intervention or (psychoeducation) control group.

*Step 5*: *Pre-intervention measurements*. After signing the informed consent form, participants fill out a second set of questionnaires that form the pre-intervention measurements. The questionnaires are all distributed online in a level 4 safe and secure environment (BankID). All questionnaires are shown in Table 3. As a compensation for the time used on completing the questionnaires the participants will receive a 200 NOK gift card for each completed assessments (pre, post, 3 M FU).

*Step 6*: *Internet-delivered intervention/control*. Participants assigned to the intervention group will start the Internet-delivered course immediately for 8 weeks, whereas the control group participants will start with the psycho-education module and receive access to the adaptive version of the intervention after 3 months. Participants will receive automatic reminders (text messages) when they are inactive in the intervention (defined as not logged in 1 days or not finished a module within 4) and will receive automatic reminders at the post-intervention and at the 3-month follow-up time points to encourage them to complete their assigned tasks and questionnaires.

**Fig. 1:**
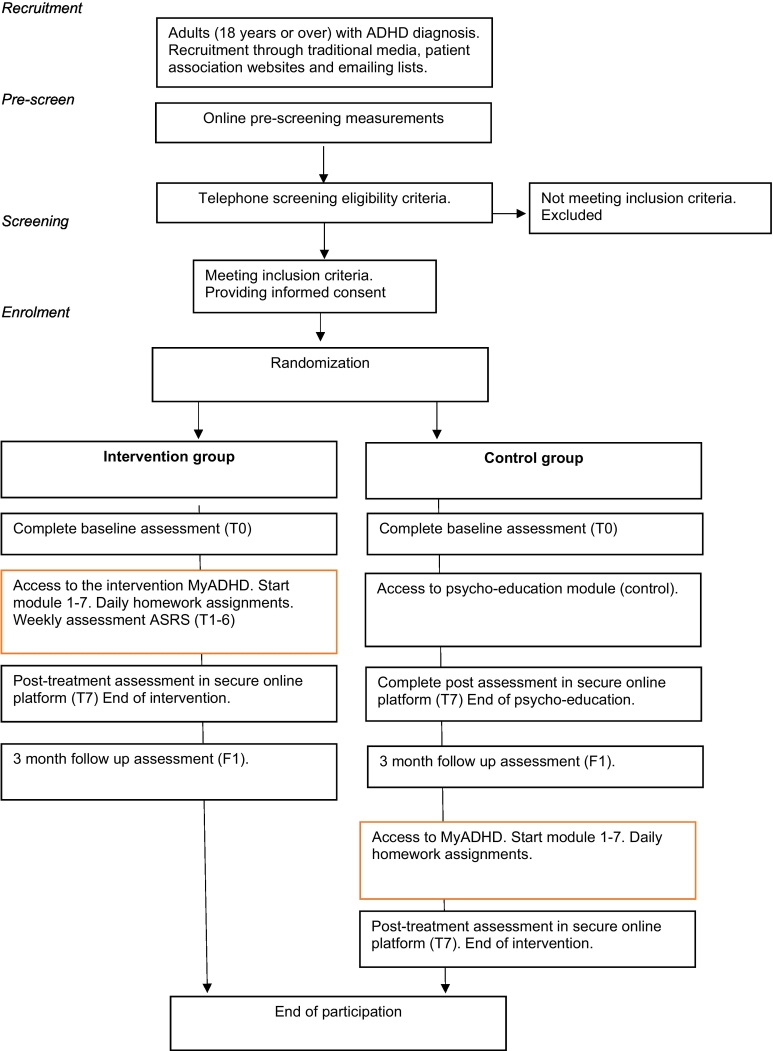
Flowchart RCT.

*Step 7*: *Control groups receives the intervention*. Participants assigned to the control group will receive access to the intervention after 3 months. For intervention group participants, the participation is complete (Fig. 1).

### 2.8 Sample size

Power calculations are informed by the feasibility study of MyADHD (Nordby et al., 2021) providing an effect corresponding to Cohens d: 0.70 on the primary outcome measure ASRS. The minimum sample size for each group (alpha set at 0.05, power at 0.95) is identified to be 45, but at least 30% more will be recruited to hedge against expected attrition (*n* = 59). The minimum total sample size will be *N* = 118. It seems realistic to recruit this in a group with an estimated prevalence of 2–3% (Franke et al., 2018).

### 2.9 Recruitment

Participants will be recruited via social media and the Norwegian patient organization that operates throughout Norway. In our feasibility study we recruited 65 participants in one week using this strategy. We therefore assume that it is feasible to recruit 118 participants within 3 months. Recruitment is planned to take place between March and May 2021.

### 2.10 Randomization

Randomization by sealed envelopes, where the research team randomly opens an envelope after a positive screen in the telephone interview and allocates the participant to the treatment regimen. Because of the nature of the intervention, trial participants and researchers cannot be blinded.

### 2.11 Data collection and management

#### 2.11.1 Data management

Data is collected via an online secure portal and by telephone assessments. The portal is a level 4 secured online Portal and data are sent encrypted to Helse Bergen's research server. Participants receive an automatic reminder once new measurements are released.

#### 2.11.2 Confidentiality

The technical infrastructure and the platform, for the Internet intervention will be made available via Youwell AS. The platform is compatible with the current GDPR. Users log on with security level 4 with BankID. The data are stored on Helse Bergen HF research server. A risk assessment of the Youwell platform has been conducted and completed at the University of Bergen.

### 2.12 Statistical methods

Analysis will be used to ensure that within subject differences and between group differences (at post and 3FU) can be calculated. IBM SPSS 21 statistical software will be used. All participants' data will be included, irrespective of treatment compliance. Descriptive statistics (Chi-square and *t-*tests) will be used to analyze sociodemographic and clinical variables at baseline of the two groups. The effect of the Internet intervention on the clinical outcome measures will be examined separately for each measure by using repeated-measures linear mixed models. Linear mixed-effects models are appropriate for analysis of longitudinal data where repeated measurements are taken from the same subjects over time and are particularly robust to missing data. The magnitude of the treatment effect within and between the two groups will be calculated using the *Cohen d* statistic. Cohen describes an effect size of 0.2 as small, 0.5 as medium, and 0.8 as large. Analyses will be conducted to assess how many participants achieved clinically significant changes at the end of the intervention and at follow-up. The assessments will be made through a comparison of pre-intervention scores with post-intervention and follow-up scores on the ASRS, the primary outcome measure in the present study. Reliable change will be assessed using the Jacobson and Truax (Jacobson and Truax, 1992) reliable change criteria.

## 3 Discussion

There is a need for the development and examination of psychological interventions tailored to the needs of adults with ADHD. There is a large evidence base for Internet-delivered interventions for common mental disorders such as anxiety (Andersson et al., 2019), depression (Karyotaki et al., 2017), and sleep problems (Zachariae et al., 2016), but not for ADHD. We expect that the results of this study will contribute to the growing research on Internet-delivered interventions and that the results will have a major impact on the management of ADHD symptoms in adults. Apart from being influenced by the core symptoms of an ADHD diagnosis - inattention, hyperactivity and impulsivity - ADHD is a clinically heterogeneous disorder were people also often experience emotional problems, procrastination, lack of motivation and low self-esteem which further impacts quality of life and day-to-day functioning (Michielsen et al., 2018; Watters et al., 2018). The MyADHD intervention, focusing on core symptoms of ADHD and their wider consequences for daily functioning, is expected to reduce the severity level of symptoms and increase quality of life for the participants.

### 3.1 Strengths

This trial will shed new light on the question whether adults with ADHD can benefit from this type of self-guided Internet-delivered interventions. This has, to the best of our knowledge, not previously been done in a large RCT, although the relevance of conducting a controlled study on Internet-delivered psychological intervention for adults with ADHD is high for a number of reasons.

As demonstrated previously, ADHD is a disorder affecting many aspects of daily life, but adults with ADHD will seldom be offered psychological help. Any intervention contributing to more people receiving better care for managing their symptoms against relatively low costs will be welcomed by all stakeholders. An additional strength of this intervention is that it combines treatment techniques that could provide help to manage the many different challenges that adults with ADHD tend to experience.

Third, the study has a control group that mimics the care as usual, where people can take medication and receive psychoeducation, i.e., the recommended first-line treatment.

Fourth, self-guided Internet-delivered interventions might be especially relevant for adults with ADHD during this pandemic. For one, with home-offices, adults are expected to independently manage work, which demands self-regulated behaviour, pandemic-related shifts to low-structure, increased stress-exposure, less support, increased family chaos and not be able to meet a therapist at a therapist office.

A final strength is the delivery over the Internet, which has the potential to reach people in need of psychological help in a larger geographical area.

### 3.2 Limitations

The current study has some limitations. First, the outcome measures are based solely on self-reports which may be subject to reporting bias, although rating scales are shown to reliably, validly and efficiently measure DSM-based ADHD symptoms in adolescent samples (Collett et al., 2003).

Second, the research study excludes those with signification personality disorders, current substance use, suicidal ideation, and other severe psychiatric problems although these patients represent a significant proportion of the population of adults with ADHD. However, since this is an entirely self-guided intervention, we needed to take these safety issues into account.

A final limitation of this study includes our sample of convenience, which may inadequately represent the full population of adults with ADHD (e.g., adults with comorbidities).

### 3.3 Implications

This study is one of the first that investigates the effects of self-guided Internet-delivered self-help in a group of adults with ADHD. Self-guided Internet-delivered interventions represent a promising possibility to offer psychological interventions to adults with ADHD, while saving expensive and scarce resources.

## Abbreviations

AAQOL-29: Adult ADHD Quality of Life Scale
ADHD: attention-deficit/hyperactivity disorder
ASRS: the adult ADHD self-rating scale
CEQ: credibility and expectancy scale
CBT: cognitive behavioral therapy
DBT: dialectical behavioral therapy
FU: follow up
GMT: goal management training
GDPR: General Data Protection Regulation
MADRS: Montgomery And Åsberg Depression Rating Scale
MADRS SR: Montgomery And Åsberg Depression Rating Scale Suicide Rating
PHQ-9: patient health questionnaire 9
PSS: perceived stress scale
T1-T7: Test time 1 – test time 7

## Trial status

Recruitment is finished and the first group of participants completed the intervention and assessments, while the second group is currently finishing the intervention and post-test measurements.

## Credit authorship contribution statement

RK is the chief investigator in this study, led the proposal and protocol development, ethics application and drafted the manuscript. AJL contributed as the domain expert in the ADHD case, to the intervention and the psychoeducation control and to the drafting of the manuscript. TN contributed as the head of INTROMAT, to the study design, and drafting of the manuscript. All authors critically reviewed and approved the final manuscript.

## Declaration of competing interest

The authors have designed and created the Internet-delivered intervention, but derive no economic profit from it.
